# The Role of HLA in the Association between IgA Deficiency and Celiac Disease

**DOI:** 10.1155/2021/8632861

**Published:** 2021-12-13

**Authors:** Dimitri Poddighe, Cristina Capittini

**Affiliations:** ^1^School of Medicine, Nazarbayev University, Nur-Sultan, Kazakhstan; ^2^Clinical Academic Department of Pediatrics, National Research Institute for Maternal and Child Health, University Medical Center, Nur-Sultan, Kazakhstan; ^3^Clinical Epidemiology and Biometric Unit, Scientific Direction, IRCCS Policlinico San Matteo Foundation, Pavia, Italy

## Abstract

Selective IgA deficiency (SIgAD) is the most frequent primary immune defect. Since SIgAD is not characterized by relevant infectious issues in most cases, it is often diagnosed during the diagnostic work up of several and different autoimmune disorders, which are associated with this primary immune defect. The genetic background of SIgAD is complex and three HLA haplotypes resulted to be more frequently associated with it; in detail, two of them include HLA-DQB1^∗^02 allelic variants, which are essential predisposing factors to develop Celiac Disease (CD). Here, we discuss the evidence regarding the role of HLA in the etiopathogenesis of SIgAD and its association with CD. Actually, the HLA region seems to play a modest role in the genetic predisposition to SIgAD and we may speculate that the association with the HLA-DQB1^∗^02 alleles (or haplotypes including them) could derive from its link with CD. Indeed, SIgAD and some related immunological alterations are likely to predispose to several autoimmune diseases (with and despite different HLA backgrounds), including CD, which is relatively common and directly associated with the HLA-DQB1^∗^02 allelic variants coding the DQ2 heterodimer. Further and specific studies are needed to make final conclusions in this regard.

## 1. Introduction

Celiac disease (CD) is a gluten-related systemic immune-mediated disorder characterized by a very variable clinical expression, including both gastrointestinal and extra-gastrointestinal manifestations. It is diagnosed by the demonstration of specific autoantibodies, such as anti-tissue transglutaminase antibody and anti-endomysium antibody (which mainly belongs to the IgA isotype), along with the presence of atrophic (small bowel) enteropathy at the histopathological level [1–2].

Selective IgA deficiency (SIgAD) is the most common primary immune deficiency worldwide [3]. Notably, SIgAD is significantly associated with CD [1], which can make the diagnosis of the latter disease be more difficult, since the main serological markers are IgA autoantibodies. Indeed, the assessment of total serum IgA concomitantly to the serological screening for CD is a mandatory test in the suspicion of CD [4–5].

The HLA (human leukocyte antigen) system represents a relevant component of the genetic predisposition to autoimmunity in general, even if the implicated loci and allelic variants are different according to the specific autoimmune disorder [6]. Notably, several studies described a significant association between SIgAD and a few HLA haplotypes [7].

In general, primary immune deficiencies and autoimmunity are linked: several autoimmune diseases may complicate the same immune deficit [8]; moreover, the diagnosis of one autoimmune disorder increases the risk of developing other autoimmune diseases and/or different autoantibodies, as it happens in CD as well [9–10].

In this review, we discuss SIgAD and CD in the perspective of the HLA system, in order to analyze and assess the specific contribution of these loci in the etiology and pathogenesis of the epidemiological association between these diseases.

## 2. Selective IgA Deficiency

Immunoglobulin A (IgA) is the most abundant antibody isotype in the human body, overall. Indeed, even though IgG have the highest blood concentration by far, IgA is also present in the mucosal surfaces of respiratory, intestinal, and genitourinary systems and then account for >70% of the total immunoglobulin pool [11]. Secretory IgA is dimeric and contributes to limit the epithelial adherence and penetration of endogenous bacteria through the mucosal surfaces, in addition to preventing infections by pathogenic microorganisms [12–13]. Serum IgA actually circulates in monomeric form, and its function in the systemic immune response has not been completely elucidated; however, it may have an immunomodulatory role, and form immune complexes with foreign antigens and clear them through the phagocytic system, but without activating the complement cascade [14–15]. However, it is clear that IgA plays a fundamental role in maintaining the homeostasis at the mucosal surfaces. In detail, secretory IgA is supposed to promote an immune exclusion by entrapping dietary antigens and microorganisms in the mucus, downregulate the expression of proinflammatory bacterial epitopes on commensal bacteria, and maintain the appropriate bacterial communities, especially in the gut [16].

Serum IgA levels are age-related: IgA is basically absent at birth and its concentration gradually increases during the pediatric age until reaching the adult levels during the adolescence (with normal levels ranging between 61 and 365 mg/dl) [17]. Total IgA deficiency is defined by serum IgA levels < 7 mg/dl. IgA deficiency is defined as partial when serum IgA levels are >7 mg/dl, but below the lower limit of the normal range according to the age [3, 17–18].

In infants and young children, low levels of serum IgA can be observed in the general context of transient hypogammaglobulinemia of infancy or their level can be selectively reduced due to delayed ontogeny of the immune system after birth in terms of IgA production. Therefore, a threshold of 4 years of age is commonly accepted to make a final diagnosis of SIgAD, which is then diagnosed in children older than 4 years, who show low IgA levels, but normal levels of IgG and IgM (in addition to normal vaccine responses and, importantly, after exclusion of secondary causes of hypogammaglobulinemia and T cell defects), even if it may be associated with IgG subclasses deficiency. Indeed, additional immunological abnormalities indicate different disorders, such as common variable immunodeficiency, secondary hypogammaglobulinemia, and unclassified antibody deficiencies [3, 19]. SIgAD is the most common immunodeficiency: worldwide, its prevalence is estimated to be around 1 : 400, despite significant variations according to the ethnicity. Indeed, it is considered more common in Caucasian populations (1 : 134–1 : 875), whereas the lowest prevalence is described in (East) Asian populations (China, 1 : 4100; Japan, 1 : 18500) [3, 20].

However, SIgAD prevalence studies are still lacking in many countries and, importantly, almost 90% of individuals with IgA deficiency have no specific symptoms or are completely asymptomatic. Overall, less than 30% of patients present with overt clinical manifestations of immunodeficiency, such as recurrent respiratory or gastrointestinal tract infections. Moreover, most patients with evident sinopulmonary infections (caused by encapsulated bacteria, such as *Haemophilus influenzae* and *Streptococcus pneumoniae*) are more likely to also have IgG subclass deficiency, especially IgG2 and IgG3 [3, 20–21]. However, an important clinical characteristic of SIgAD is its frequent association with allergy and autoimmunity, which may be the only “clinical manifestation” of this primary immune defect [19].

## 3. Allergy and Autoimmunity in Selective IgA Deficiency

A wide range of allergic disorders (including allergic conjunctivitis, rhinitis, urticaria, eczema, food allergy, and asthma) are often diagnosed in SIgAD patients [3, 22]. In a recent report, allergy was evidenced in 84% of patients with SIgAD (age range: 4–32 years) [23]. However, although a significant epidemiological association is supported by most studies on this topic, the actual prevalence of allergy among SIgAD patients is debated and may vary according to several factors, including the ethnical background [24]. In practice, allergic manifestations are the presenting symptoms in at least 25–50% of SIgAD patients [18, 21]. It is speculated that IgA deficiency by itself may bring to an increased prevalence of allergic disorders. Indeed, IgE concentrations are often increased in patients with SIgAD (and, in detail, atopic children), which may be due to a compensatory mechanism for a low secretory IgA level. Conversely, reduced IgA to gastrointestinal antigens were described in the mucosa of atopic children, which led to the hypothesis that gut luminal IgA deficiency may promote eczema and food allergy [25–27].

Similarly, a number of autoimmune diseases are associated with SIgAD. Indeed, according to different studies, at least 5–30% of SIgAD patients are diagnosed with concomitant autoimmune disorders, including idiopathic thrombocytopenic purpura, Graves' disease, autoimmune hemolytic anemia, type 1 diabetes mellitus, rheumatoid arthritis, thyroiditis, systemic lupus erythematosus (SLE), autoimmune hepatitis, and CD [21, 28–29].

The pathogenesis of this relationship between SIgAD and autoimmunity is not completely understood. However, considering the number of different autoimmune disorders linked to SIgAD, multiple mechanisms could be variably implicated to explain this link. Odineal et al. recently summarized the potential mechanisms involved in SIgAD-related autoimmunity [30]. As mentioned, serum IgA can bind antigens and clear them without activating the complement and, thus, limiting the inflammatory responses: accordingly, IgA deficit may predispose the immune system to become sensitized to autoantigens through mechanisms of molecular mimicry [7, 31]. In this regard, the concomitant deficit of mucosal IgA can expose the adaptive immune system to some pathogenic or commensal microorganisms, promoting cellular and humoral responses that may cross-react with self-antigens [3, 30].

Moreover, SIgAD definitely recognizes a background of genetic predisposition, which is heterogenous and not well defined, yet. Mutations in transmembrane activator and calcium modulator and cyclophilin ligand interactor (TACI, TNFRSF13B) have been found in a subset of patients with IgA deficiency, but also in patients with common variable immunodeficiency (CVID). Even though the pathogenic role TACI mutations in SIgAD is controversial, it is clear that SIgAD can be associated with B cell, T cell, or cytokine abnormalities, which in turn may be implicated in the susceptibility to autoimmunity [30, 32–34].

Finally, the potential role of the HLA system was also considered, which may favor the development of SIgAD and concomitantly predispose to autoimmunity. As discussed later, specific HLA haplotypes resulted to be associated with SIgAD, and some HLA allelic variants also appeared to be independently associated with several autoimmune diseases, including SLE, CD and dermatitis herpetiformis, type I insulin-dependent diabetes, myasthenia gravis, and scleroderma [27, 30].

## 4. The HLA System in Selective IgA Deficiency

SIgAD usually occurs sporadically, but familial cases are described, even though no Mendelian inheritance pattern can be clearly defined. Indeed, the pedigrees of IgA-deficient-related individuals can be both autosomal recessive and autosomal dominant [18]. However, the genetic component appears to be relevant: the risk of developing SIgAD can be up to 50-fold higher in first-degree family members of patients with SIgAD compared to the general population. Moreover, this risk is 4-fold greater when the affected parent is the mother compared to the father [34–35]. In summary, SIgAD is likely to recognize a multifactorial etiology with a multigenic inheritance, where epigenetic aspects also play a role.

In such a not well-defined genetic background, the HLA loci have been investigated to understand if they could play a direct role in the pathogenesis of the SIgAD. Indeed, specific HLA haplotypes, including both class I and II HLA genes, were found to be more frequently represented in patients with SIgAD [3]. In detail, three major haplotypes resulted to be associated with SIgAD. HLA-B^∗^0801/DRB1^∗^03/DQB1^∗^0201 was strongly associated with SIgAD in Caucasian populations, especially in Northern Europe (Sweden, Norway, Iceland, Finland, and Germany) [36–37]. Indeed, a 13% prevalence of SIgAD was initially reported in individuals who are homozygous for the HLA B8/DR3 haplotype, corresponding to an extremely high relative risk (RR = 77.8) [38]. However, eventual and larger studies evidenced a much more modest effect (RR = 11.1 for homozygosity, RR = 3.4 for heterozygosity) of this haplotype on the risk for SIgAD than what was previously suggested [39]. Interestingly, some authors suggested that the SIgAD association with this haplotype could have been actually due to a class III HLA region allele, based on a study comparing this haplotype across SIgAD patients from Sardinia (Italy), North Europe, Australia, and USA [38, 40–42]. A second haplotype (HLA-DRB1^∗^0701/DQB1^∗^0202) has been associated with SIgAD, again in Northern Europe, whereas a third haplotype (HLA-DRB1^∗^0102, DQB1^∗^0501) has been described more frequently in Southern Europe (Spain and Italy) and in Southwest Asia (in detail, Iran) [37, 43–45]. Conversely, some authors explained the low prevalence of SIgAD in Chinese population with the lower frequency of these disease-related haplotypes/alleles in China [46].

Despite the number of studies describing the increased frequency of these haplotypes in SIgAD patients, a recent genetic analysis showed that the influence of HLA in SIgAD genetics is likely to be modest, and suggested that other non-HLA genes and/or other epigenetic influences from environmental factors may be more relevant for the development of SIgAD. However, at the same time, these researchers observed that some specific HLA allelic variants may have some influence on the IgA serum levels. For instance, HLA-A^∗^01 and HLA-B^∗^14 alleles were associated with an increased IgAD risk and carriers resulted to have a significantly lower mean serum IgA concentration; conversely, HLA alleles B^∗^07 and DRB1^∗^15 were found to confer protection against SIgAD and, accordingly, carriers showed a significantly increased mean serum IgA concentration [47].

Notably, a recent study proposed an “epigenetic” role for the HLA region. A specific micro-RNA (miR-6891-5p), which is encoded by an intronic sequence inside HLA-B, resulted to regulate the expression of the immunoglobulin heavy chain alpha 1 and 2 (IGHA1 and IGHA2) genes at the post-transcriptional level, thus potentially affect IgA levels and contribute to the development of SIgAD [48].

Therefore, non-HLA loci seem to be as important as—or actually more than—HLA genes in the determination of the genetic susceptibility to SIgAD. Recent studies proposed associations with several non-HLA loci (e.g., CLEC16A, CTLA4, ICOS, FAS, IL6, and IL10), but conclusive evidence for their role in the pathogenesis of SIgAD is still lacking [49].

## 5. HLA-DQ Genes in Selective IgA Deficiency and Celiac Disease

The prevalence of SIgAD in CD patients is estimated to be around 1 : 40 (2–2.5%). Indeed, IgA levels should be systematically measured in patients diagnosed with CD and, even earlier, during the diagnostic work up for CD, considering the implication of low serum IgA levels for the reliability of CD serological tests based on the detection of specific IgA autoantibodies, such as anti-tTG, EMA, anti-gliadin antibody, and antibody to deamidated gliadin peptides [5, 50–51].

Similarly, CD is more frequent in children with SIgAD than in the general population and, actually, their association looks even stronger in this direction. Meini et al. reported a 7.7% prevalence in children affected with SIgAD [52]. In other studies, CD prevalence reached values of 15–30%, when SIgAD patients had been already diagnosed with other autoimmune disorders [53–54]. Another study by Lenhardt et al. confirmed a similar prevalence of CD (8.7%, *n* = 11 CD patients) in their cohort of 126 patients with SIgAD (age range: 2–20 years). Additionally, these authors also described the HLA-DQ genetic background of these patients (DQ2 : *n* = 9, DQ8 : *n* = 2) [55].

As mentioned, the necessary environmental trigger for CD is well known, namely, the dietary exposure to gluten. Indeed, the pathogenesis of CD can be summarized as a gluten-induced activation of the adaptive immune response: gluten-reactive T lymphocytes are found in the lamina propria, which display a Th1 phenotype with a cytokine production dominated by IFN-*γ*, even though gliadin-specific Th17 cells and CD8+ T cells have been described, too [56].

A key finding supporting the central role of the adaptive immune response in CD pathogenesis is the constant association with specific HLA class II molecules. Indeed, CD is strongly associated with the carriage of DQ2 and/or DQ8 MHC heterodimers. In detail, almost 100% of CD patients carry the specific HLA alleles DQA1^∗^0501-DQB1^∗^02 (coding the DQ2 MHC heterodimer) and/or DQA1^∗^0301-DQB1^∗^0302 (coding the DQ8 MHC heterodimer) [1–2]. Among these HLA-DQ genes, recent studies showed the epidemiological importance of HLA-DQB1^∗^02 alleles in the pediatric CD population [57–58]. In detail, our group highlighted that around or >95% of CD patients (and especially children) carry at least one copy of HLA-DQB1^∗^02 variants [59–61]. However, such an HLA immunogenetic predisposition to CD is quite common in the general population (since 30%–40% of the individuals in Europe, North America, and other populations have been demonstrated to carry HLA-DQB1^∗^02 alleles) and, thus, it is not sufficient for developing CD: indeed, only a minority of these MHC DQ2/DQ8 carriers (around 3%) actually develop CD during life, despite a comparable dietary exposure to gluten [62–64].

Interestingly, the main SIgAD-associated HLA haplotypes (HLA-B^∗^0801/DRB1^∗^03/DQB1^∗^0201 and HLA-DRB1^∗^0701/DQB1^∗^0202) included the allelic variants coding for the MHC-DQ2 heterodimer. In detail, 45% of SIgAD patients have the haplotype 8.1 (HLA-A1, B8, DR3, and DQ2) compared to 16% of the general population [39, 65]. These HLA-DQ genes may concomitantly favor the development of SIgAD and predispose to CD. Even though the most recent evidence seems to reappraise the role of HLA in the pathogenesis of SIgAD, some correlations between a few HLA alleles and the level of serum IgA were actually observed, as previously explained [47].

Moreover, SIgAD could be a risk factor for CD regardless of the common HLA genetic background, through a series of immunopathogenic mechanisms. In detail, the low levels of secretory IgA to protect mucosal barriers could increase the exposure to pathogens and foreign antigens. Also, IgA may also play a regulatory role in the general homeostasis of the immune system: T regulatory cell deficiency was evidenced in 64% of SIgAD patients, and a number of alterations of (memory) B cells were described in these patients, all of which may potentially contribute to autoimmunity [66–69]. In this sense, SIgAD may favor the gluten sensitization in patients who are HLA-predisposed to mount an immune response against gluten-derived peptides. Then, the association between SIgAD and HLA haplotypes, including DQB1^∗^02 alleles, may indirectly result from the pathogenetic role of SIgAD by itself in CD development, considering the greater prevalence of CD compared to other autoimmune disorders and its strong and direct association with HLA-DQB1^∗^02 alleles.

Actually, the concept of SIgAD itself as a risk factor for CD and, in general, for autoimmune diseases, appears to be more likely than a general association between SIgAD and autoimmunity based on a common HLA genetic background, which should concomitantly promote both SIgAD and CD or other autoimmune disorders. Indeed, SIgAD has been described in numerous and very diverse autoimmune diseases, which differ in terms of immunopathogenic mechanisms and HLA predisposition [7, 30]. For instance, the prevalence of SIgAD among children with juvenile idiopathic arthritis (JIA), which is one of the most frequent rheumatic disorders in children, was reported to range from 1 to 4.35% (weighted average of 2.7%) [30], which is as significant as the frequency of SIgAD in CD patients. However, the HLA genetic predisposition in JIA is variable and not much linked to HLA-DQ alleles [70–71].

Moreover, the recent advances in the understanding of the interplay between gut microbiota and immune system suggested that IgA may contribute to the establishment and maintenance of beneficial interactions with the microbiota [72]. Therefore, SIgAD may affect the microbiota composition in the gut, and that may be an additional mechanism for such a strong association between SIgAD and CD, considering the growing evidence that supports the role of microbiota in the pathogenesis of several autoimmune disorders [30]. Very recently, spontaneous inflammation in the ileum (but not the other parts of the gastrointestinal tract) was described in IgA−/− mice, which was also associated with skewed intestinal microbiota composition [73]. In the human counterpart, Moll et al. described a perturbed microbiota in individuals affected with SIgAD, which resulted to be enriched of species with increased proinflammatory potential [74]. Previously, other studies suggested a critical and nonredundant role of IgA in controlling gut microbiota composition in humans and maintaining a diverse and stable gut microbial community, even though there were differences in terms of phyla-relative abundance and diversity in SIgAD patients across these studies [75–77].

Even though no clear “celiac” signature has been identified in the microbiome of CD patients, the lack of secretory IgA is likely to alter the mucosal homeostasis of the local microbiota along the gastrointestinal tract [78–80]. In the small bowel, the alterations of the gut microbiota could perturb the mucosal barrier, impair its permeability to antigens, and finally promote immunological phenomena of cross-reactivity [81–82]. Modifications of the salivary and gut microbiome could affect the digestion of nutrients (including gluten proteins) and, thus, their ability to be recognized by the immune system and trigger the immunopathological events leading to CD [83–84].

In this regard, it is also worth to mention that several studies highlighted the potential and direct role of HLA-DQB1 in driving the gut microbial colonization process. De Palma et al. first investigated a cohort of newborns and infants being first-degree relatives of CD patients: they were analyzed for their HLA class II (DQA1 and DQB1) genotype. They found an association between higher proportions of the *Bacteroides-Prevotella* group and the high genetic risk group, basically represented by those individuals being DQB1^∗^02 homozygous or double heterozygous for DQB1^∗^02 and DQB1^∗^0301. Total Gram-negative bacteria and *E. coli*, *Streptococcus-Lactococcus* spp., the *E. rectale-C. coccoides* group, *C. lituseburense*, and the *C. histolyticum* group proportions followed a similar trend when comparing the high- versus the low-genetic risk groups [85].

The larger PROFICEL study further supported this concept, in addition to investigating the concomitant contribution of breastfeeding. Indeed, specific features of fecal microbiota were associated with the genetic risk of developing CD, based on the HLA-DQ genotype, regardless of the milk-feeding type. In detail, the authors here described an increased number of *Bifidobacterium* spp. and *B. longum* in the microbiota of infants with the lowest genetic risk, whereas increased numbers of bacteria belonging to the *Staphylococcus* spp. and *B. fragilis* group were observed in infants with the highest genetic CD risk [86].

A more recent study also supported the hypothesis that a reduced abundance of *B. longum*, dependent on both genetic (also HLA-related) and environmental factors, may favor CD development. Additionally, this study evidenced a faster reduction in secretory IgA fecal levels in children who developed CD over time compared to healthy ones: this might suggest that a premature reduction of secretory IgA levels in the group of CD children could be related to shifts in bacterial community development, which in turn may affect the maturation of the mucosal immune functions, possibly increasing the risk for autoimmune dysfunctions as well [87]. Indeed, in a previous study, reduced IgA-coated bacteria in CD patients were associated with intestinal dysbiosis [88].

## 6. Conclusion

**Figure 1:**
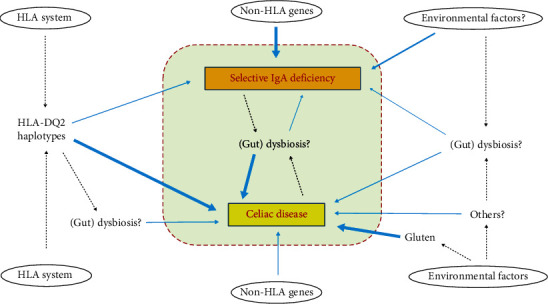
Schematic overview of the etiologic factors and aspects that are implicated in the pathogenesis of SIgAD and CD and may variably interplay to explain the association between these two diseases. HLA-DQ2 allelic variants are the necessary genetic background in CD patients and are also associated in part of SIgAD patients. Non-HLA genes (such as TACI, TNFRSF13B, CLEC16A, CTLA4, ICOS, FAS, IL-6, and IL-10) seems to mainly contribute to the genetic predisposition to SIgAD. A number of environmental factors are supposed to be implicated in both diseases; however, these are not well defined, except for dietary gluten exposure, which is a mandatory condition for developing CD. In addition to a direct role, all these factors might impact on the risk of developing CD and/or SIgAD by affecting (gut) microbiome; the potential dysbiosis associated with each disease might also contribute to pathogenesis of the other one.

Several aspects and mechanisms can be theoretically implicated in the association between CD and SIgAD, including the HLA system (in detail, HLA-DQ2-related allelic variants), non-HLA genes, and environmental factors, as schematically summarized in Figure 1.

Despite a number of studies describing the association between a few HLA haplotypes and SIgAD, the most recent evidence suggested that the direct influence of HLA genes in its pathogenesis is likely to be modest, supporting a heterogeneous genetic background in the context of an etiologic and pathogenic picture where non-HLA genes and/or epigenetic influences from environmental factors play a relevant role for the development of SIgAD.

The two main haplotypes associated with SIgAD both include HLA-DQB1^∗^02 alleles, which are known to be the genetic predisposing factor to CD in >90% of patients. The prevalence of SIgAD in CD patients is around 2–2.5%, whereas pediatric studies show up to 10% prevalence of CD in SIgAD patients. We may speculate that such an association between SIgAD and HLA-DQB1^∗^02 could be driven by the higher population prevalence of CD compared to other SIgAD-associated immune diseases, all of which may recognize a direct pathogenic contribution from low blood/mucosal levels of IgA. However, some influence of HLA genes and, in detail, HLA-DQB1^∗^02 alleles on the development of SIgAD (maybe through microbiome alterations and related epigenetic/immunological mechanisms) cannot be definitely ruled out. Further and specific studies are needed to make final conclusions in this regard.
